# Supplementary material for: Development of 99mTc-Hynic-Adh-1 Molecular Probe Specifically Targeting N-Cadherin and Its Preliminary Experimental Study in Monitoring Drug Resistance of Non-Small-Cell Lung Cancer
Source: Cancers (Basel). 2023 Jan 26;15(3):755. doi: 10.3390/cancers15030755 (PMC9913320; doi:10.3390/cancers15030755)
Supplement: Supplementary file 1 [file cancers-15-00755-s001.zip › cancers-2140273-supplementary.pdf]

Supporting materials

1. Identification of Cy3-ADH-1

Through the solid phase synthesis method, Cy3-ADH-1 is a red powder, with a purity of 99.12% by HPLC analysis and a main peak molecular weight of 1226.45 (theoretical molecular weight of Cy3-ADH-1 1225.71) identified by mass spectrometry analysis (Figure S1–2).

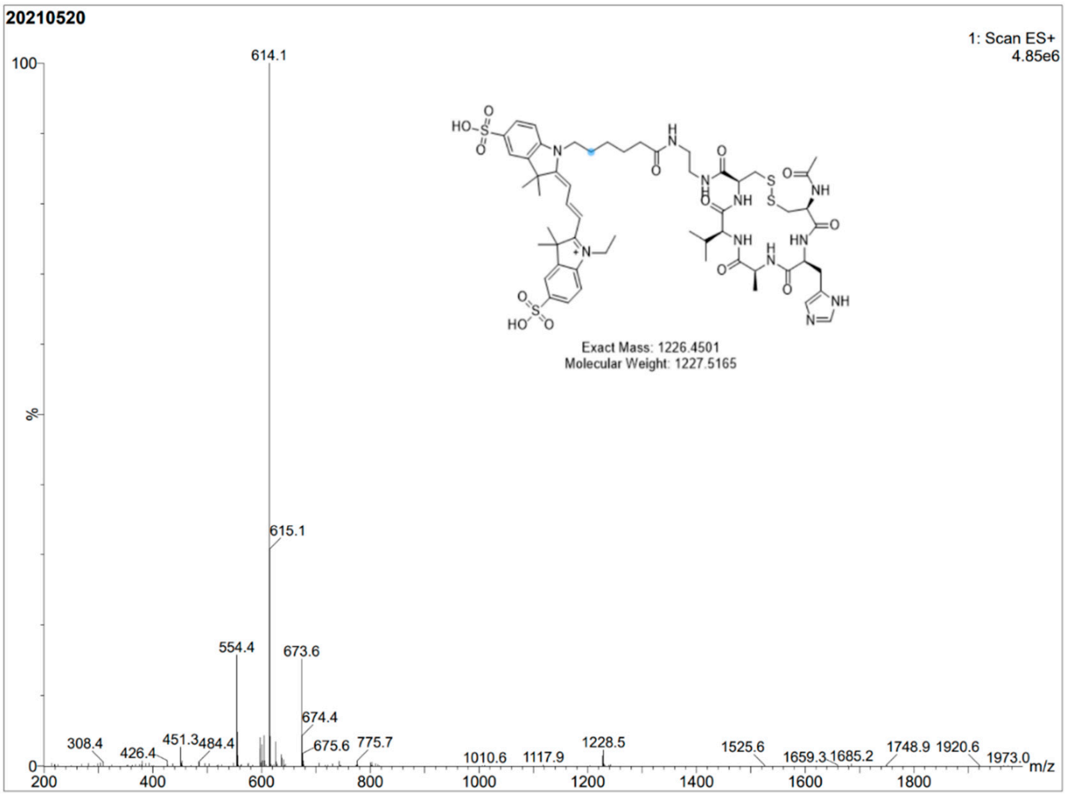

Figure S1. Mass chromatogram of Cy3-ADH-1. The m/z of the main peak is 1226.45.

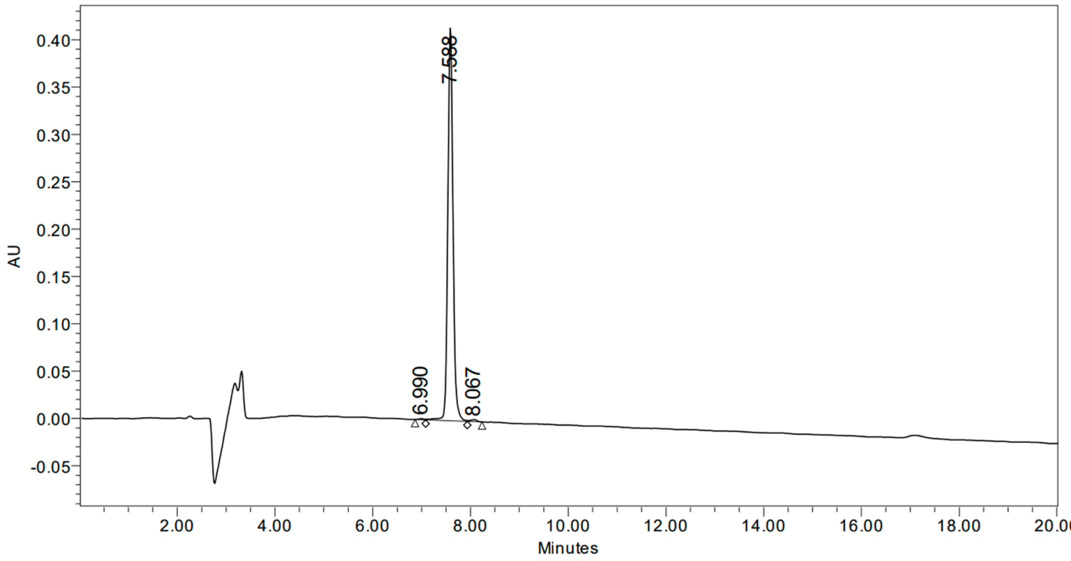

Figure S2. Cy3-ADH-1 spectrum analyzed by high performance liquid chromatography analyzer.

2. Identification of Cy7-ADH-1

Cy7-ADH-1 is a blue powder with a purity of 98.89% by HPLC analysis and a main peak molecular weight of 1277.47(theoretical molecular weight of Cy7-ADH-1 1278.59) identified by mass spectrometry analysis (Figure S3–S4).

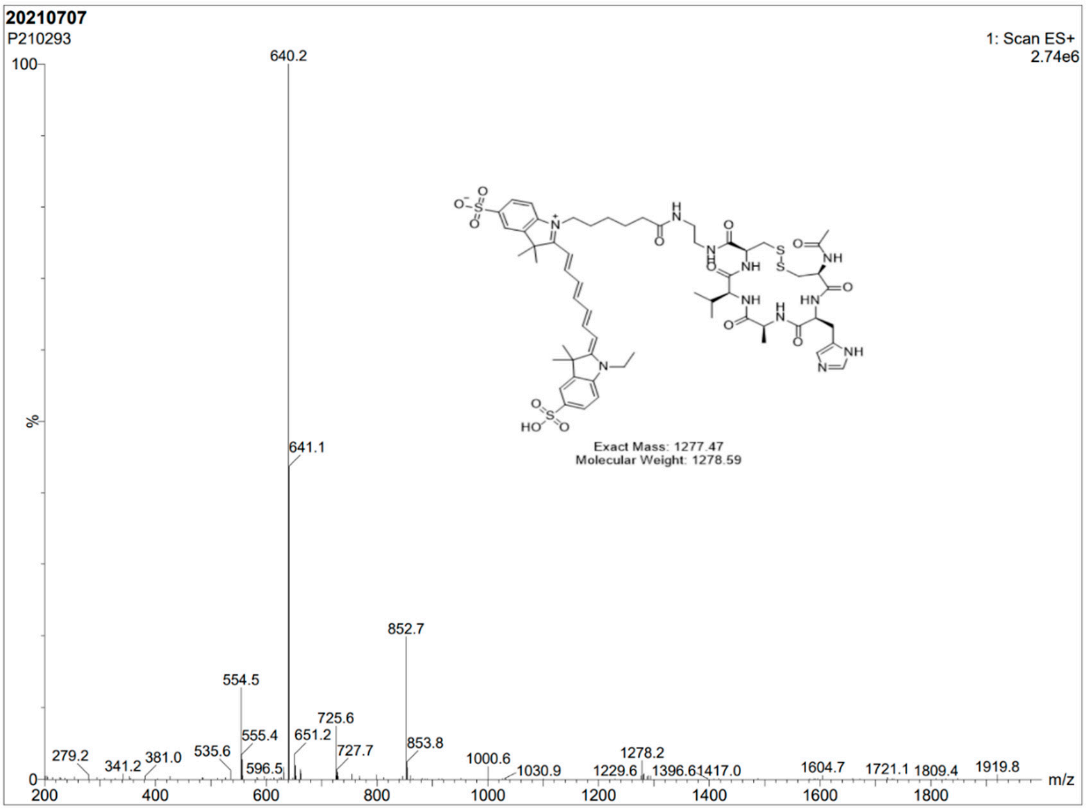

Figure S3. Mass chromatogram of Cy7-ADH-1. The m/z of the main peak is1277.47.

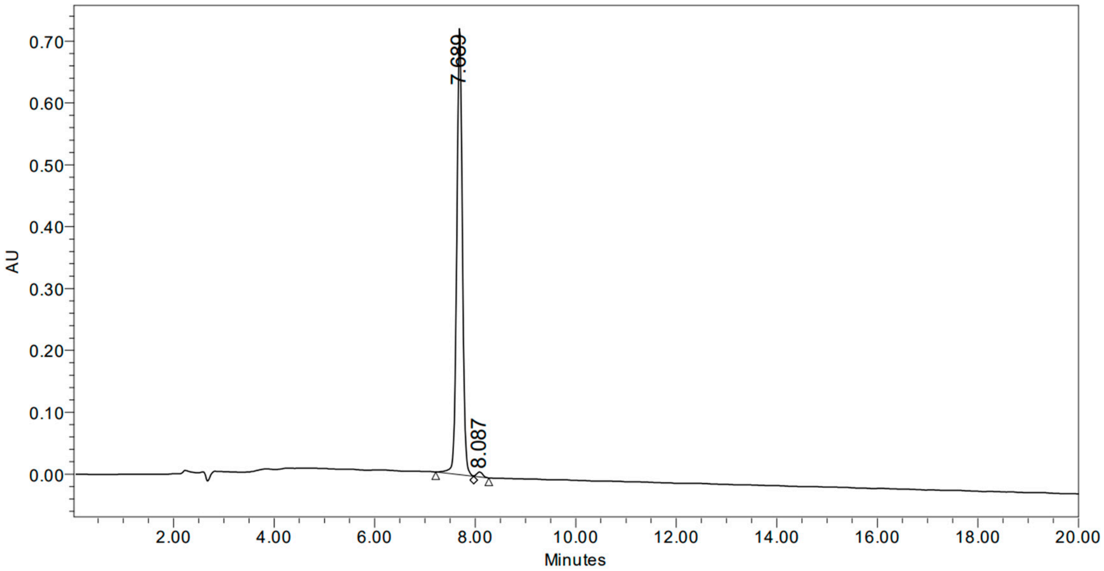

Figure S4. Cy7-ADH-1 spectrum analyzed by high performance liquid chromatography analyzer.

3. Identification of ADH-1

Through the solid phase synthesis method, ADH-1 is a pink powder with a purity of 98.76% by HPLC analysis. The main peak molecular weight identified by mass spectrometry analysis is 613.25, which is consistent with the theoretical molecular weight 613.75 of ADH-1 (Figure S5–S6).

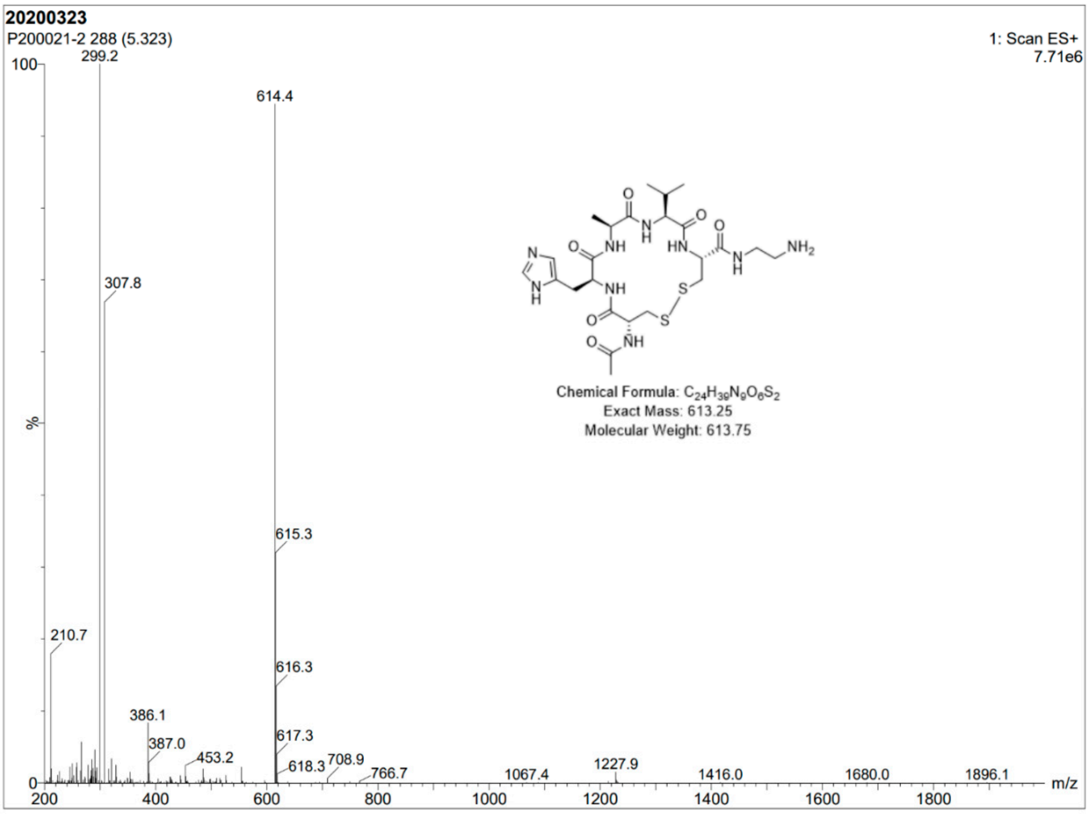

Figure S5. Mass chromatogram of ADH-1. The m/z of the main peak is 613.25.

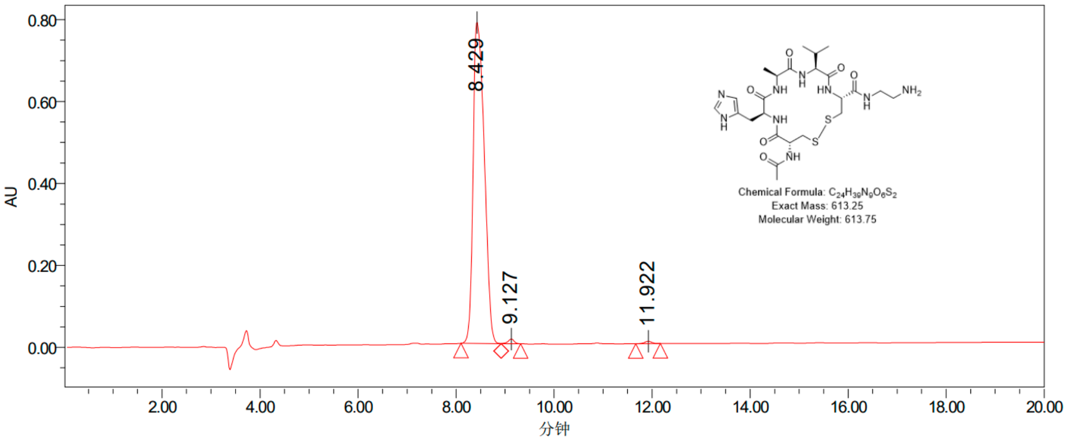

Figure S6. ADH-1 spectrum analyzed by high performance liquid chromatography analyzer.

4. Precursor HYNIC-ADH-1 synthesis

The synthesized HYNIC-ADH-1 is a white powder with a purity of 98.83% by HPLC analysis and a main peak molecular weight of 748 (theoretical molecular weight of HYNIC-ADH-1 is 748.29) identified by mass spectrometry analysis (Figure S7–S8).

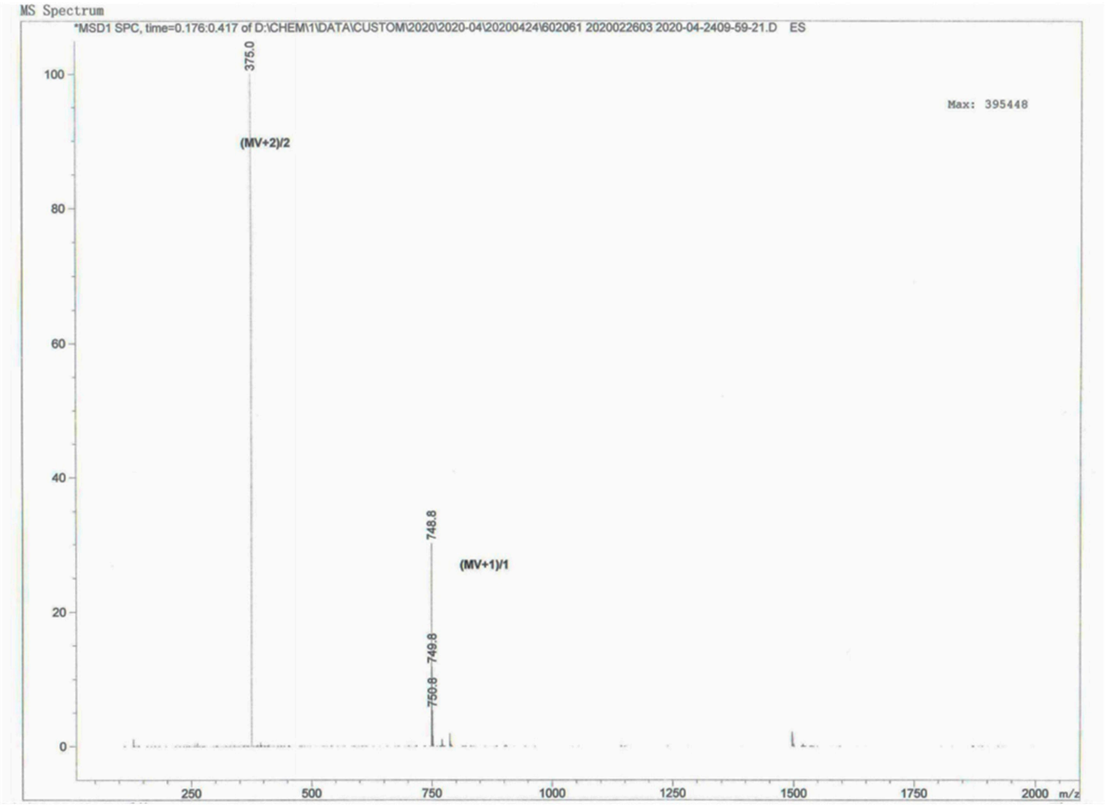

Figure S7. Mass chromatogram of HYNIC-ADH-1. The m/z of the main peak is 898.38, in the accordance with the MWt of 899.05.

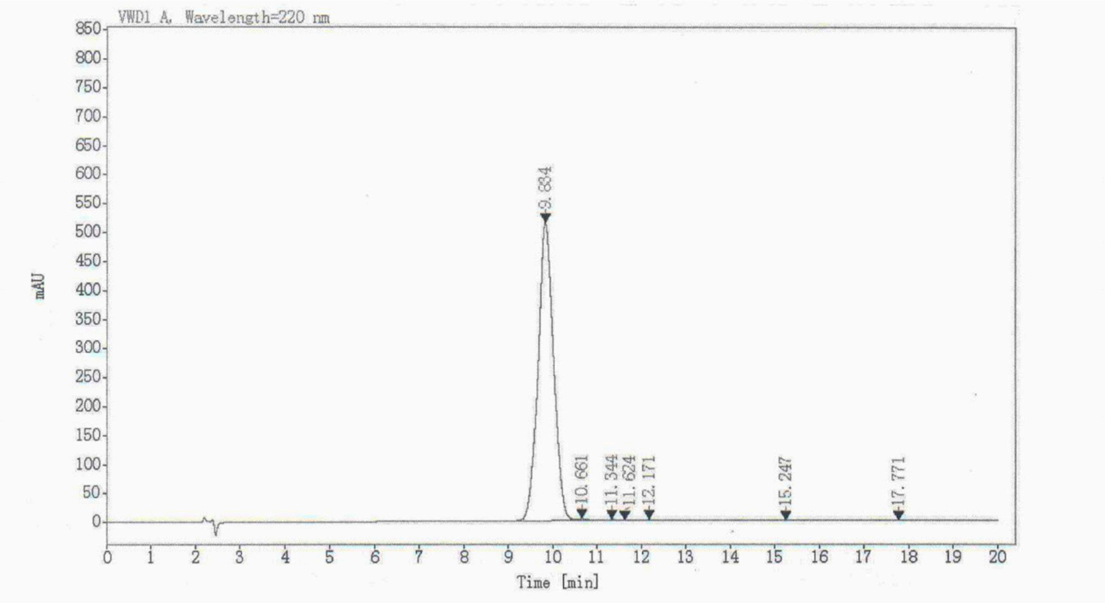

Figure S8. HYNIC-ADH-1 spectrum analyzed by high performance liquid chromatography analyzer.

5. Fluorescence Staining

Figure S9 shows the in vitro binding of Cy3-ADH-1 with different concentrations to the two cell lines under fluorescence microscopy. The in vitro binding of Cy3-ADH-1 to the two cell lines at different times under fluorescence microscope is shown in Figure S10.

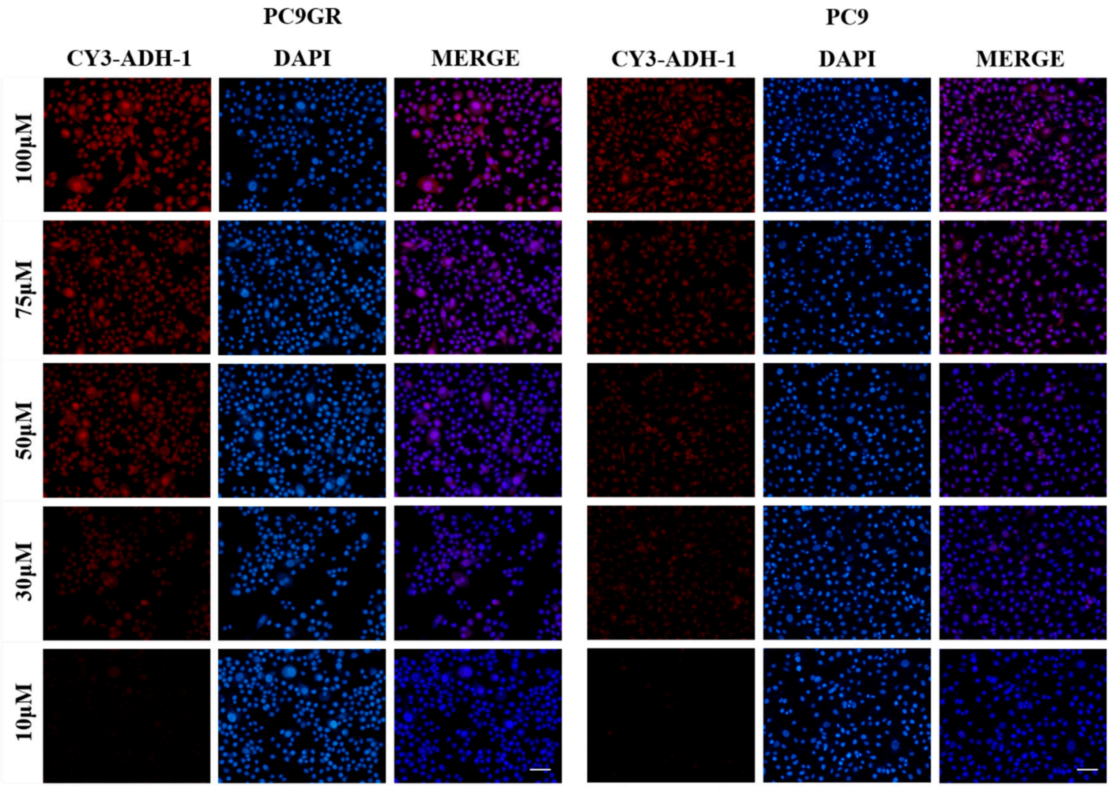

Figure S9. In vitro binding of Cy3-ADH-1 to PC9GR and PC9 visualized using fluorescence microscope (100×), increasing uptake of Cy3-ADH-1 with increasing incubation concentrations. Scale bar, 100 μm.

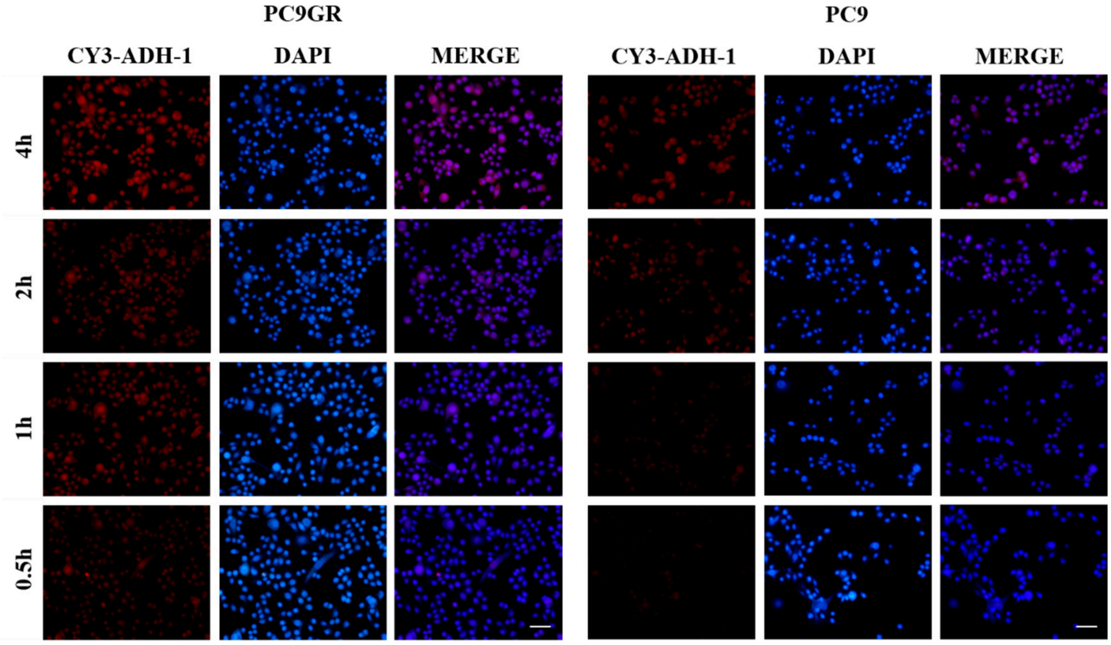

Figure S10. In vitro binding of Cy3-ADH-1 to PC9GR and PC9 visualized using fluorescence microscope (100×), increasing uptake of Cy3-ADH-1 with increasing incubation times. Scale bar, 100 μm.

6. Biodistribution studies

In vivo biodistribution studies in normal C57/BL6 mice and Xenograft-bearing nude mice are shown in Table S1.

Table S1. Biodistribution profile of ^{99m}Tc-HYNIC-ADH-1 in normal C57/BL6 mice and Xenograft-bearing nude mice. Values are expressed as %ID/g (x ± SD, n=4).

Tissue	Normal C57/BL6 mice				Xenograft-bearing nude mice			
	30 min	60 min	120 min	240 min	30 min	60 min	120 min	240 min
Brain	0.18 ± 0.05	0.09 ± 0.02	0.10 ± 0.02	0.08 ± 0.05	0.05 ± 0.01	0.07 ± 0.01	0.05 ± 0.00	0.03 ± 0.00
Heart	1.71 ± 0.30	0.49 ± 0.29	0.43 ± 0.07	0.32 ± 0.04	0.57 ± 0.03	0.42 ± 0.08	0.28 ± 0.01	0.23 ± 0.01
Lung	3.83 ± 0.12	1.70 ± 0.14	1.54 ± 0.56	1.08 ± 0.27	1.50 ± 0.04	1.20 ± 0.16	0.68 ± 0.11	0.56 ± 0.08
Liver	1.95 ± 0.17	1.06 ± 0.20	0.86 ± 0.15	0.68 ± 0.12	0.76 ± 0.06	0.66 ± 0.06	0.59 ± 0.01	0.55 ± 0.10
Spleen	1.25 ± 0.10	0.73 ± 0.24	0.53 ± 0.12	0.45 ± 0.11	0.35 ± 0.03	0.27 ± 0.02	0.22 ± 0.02	0.20 ± 0.01
Kidney	12.10 ± 0.47	9.01 ± 0.76	6.46 ± 0.30	6.16 ± 0.51	3.77 ± 1.00	2.85 ± 0.54	3.13 ± 0.70	3.13 ± 0.54
Stomach	2.24 ± 0.42	0.94 ± 0.23	0.66 ± 0.09	1.28 ± 0.43	1.16 ± 0.18	0.78 ± 0.32	0.73 ± 0.31	0.69 ± 0.29
Intestine	2.96 ± 0.30	2.14 ± 0.43	1.78 ± 0.20	1.26 ± 0.33	0.43 ± 0.03	1.38 ± 1.01	0.58 ± 0.12	0.88 ± 0.38
Muscle	1.55 ± 0.80	0.44 ± 0.08	0.52 ± 0.29	0.42 ± 0.21	0.50 ± 0.47	0.47 ± 0.10	0.24 ± 0.01	0.25 ± 0.04
Bone	1.45 ± 0.11	0.61 ± 0.14	0.58 ± 0.30	0.44 ± 0.15	0.46 ± 0.03	0.37 ± 0.04	0.26 ± 0.04	0.21 ± 0.01
Skin	3.45 ± 0.44	1.90 ± 0.58	1.69 ± 0.40	1.05 ± 0.18	1.22 ± 0.05	1.33 ± 0.33	1.21 ± 0.44	1.73 ± 0.08
Blood	3.31 ± 0.37	1.23 ± 0.07	0.84 ± 0.19	0.58 ± 0.08	1.35 ± 0.18	0.84 ± 0.03	0.49 ± 0.05	0.48 ± 0.20
T(PC9)	-	-	-	-	0.99 ± 0.15	0.91 ± 0.03	0.79 ± 0.06	0.64 ± 0.16
T(PC9GR)	-	-	-	-	1.09 ± 0.15	1.26 ± 0.10	1.32 ± 0.08	0.75 ± 0.19
